# X-Ray Fluorescence Imaging: A New Tool for Studying Manganese Neurotoxicity

**DOI:** 10.1371/journal.pone.0048899

**Published:** 2012-11-19

**Authors:** Gregory Robison, Taisiya Zakharova, Sherleen Fu, Wendy Jiang, Rachael Fulper, Raul Barrea, Matthew A. Marcus, Wei Zheng, Yulia Pushkar

**Affiliations:** 1 Department of Physics, Purdue University, West Lafayette, Indiana, United States of America; 2 School of Health Sciences, Purdue University, West Lafayette, Indiana, United States of America; 3 Advanced Photon Source, Argonne National Laboratory, Argonne, Illinois, United States of America; 4 Advanced Light Source, Lawrence Berkeley National Laboratory, Berkeley, California, United States of America; University of Melbourne, Australia

## Abstract

The neurotoxic effect of manganese (Mn) establishes itself in a condition known as *manganism* or Mn induced parkinsonism. While this condition was first diagnosed about 170 years ago, the mechanism of the neurotoxic action of Mn remains unknown. Moreover, the possibility that Mn exposure combined with other genetic and environmental factors can contribute to the development of Parkinson's disease has been discussed in the literature and several epidemiological studies have demonstrated a correlation between Mn exposure and an elevated risk of Parkinson's disease. Here, we introduce X-ray fluorescence imaging as a new quantitative tool for analysis of the Mn distribution in the brain with high spatial resolution. The animal model employed mimics deficits observed in affected human subjects. The obtained maps of Mn distribution in the brain demonstrate the highest Mn content in the globus pallidus, the thalamus, and the substantia nigra pars compacta. To test the hypothesis that Mn transport into/distribution within brain cells mimics that of other biologically relevant metal ions, such as iron, copper, or zinc, their distributions were compared. It was demonstrated that the Mn distribution does not follow the distributions of any of these metals in the brain. The majority of Mn in the brain was shown to occur in the mobile state, confirming the relevance of the chelation therapy currently used to treat Mn intoxication. In cells with accumulated Mn, it can cause neurotoxic action by affecting the mitochondrial respiratory chain. This can result in increased susceptibility of the neurons of the globus pallidus, thalamus, and substantia nigra pars compacta to various environmental or genetic insults. The obtained data is the first demonstration of Mn accumulation in the substantia nigra pars compacta, and thus, can represent a link between Mn exposure and its potential effects for development of Parkinson's disease.

## Introduction

Manganese (Mn) is an essential element required in trace amounts for proper body function. However, despite its vital role in enzymatic reactions, excessive Mn exposure leads to a condition known as manganism first noted by Couper in 1837 [1]. Clinical signs and symptoms of manganism closely resemble those of Parkinson's disease (PD) [2], and both diseases are pathologically associated with damage to the basal ganglia [3], [4]. PD and early stage manganism express different clinical symptoms, such as an amelioration/no improvement of symptoms when undergoing levodopa therapy, resting/kinetic tremors, and asymmetry/symmetry of clinical signs, respectively [5]. Although some studies report the reversal of symptoms for early-stage manganism, studies of advanced manganism show continued progression of the disease after ceasing Mn exposure [2]. The commonality of symptoms shared between PD and advanced manganism suggests a potential relationship between the diseases; see the review articles [2], [6]. For this reason, various epidemiological studies were designed to examine the correlation between increased occupational and/or environmental exposure to Mn and incidence of PD [7]–[15]. Recent review on epidemiology of PD [16] admitted that evidences are still inadequate to conclude about the role of Mn as environmental factor in PD.

Knowledge of Mn distribution in various brain structures provides fundamental information for understanding the transport and neurotoxic effects of Mn. Mn crosses the brain-barrier system (BBB) with an influx into choroid plexus (CP) cells of the blood-cerebrospinal fluid barrier about 100 times greater than into endothelial cells of the BBB [17], [18]. Areas of the brain which do not have a BBB, such as circumventricular organs, quickly absorb Mn while uptake into other brain areas is limited by the BBB. Information regarding Mn transport into brain cells remains incomplete. Multiple studies suggest that Mn utilizes transporters primarily reserved for other biologically relevant transition metals and that an elevated Mn intake may disrupt the homeostasis of these essential metal ions via competition for inter- or intra- cellular pathways [9], [19]–[21]. Possible pathways for Mn transport include binding to transferrin to initiate receptor-mediated endocytosis and direct transport through the cell membrane via the divalent metal transporter 1, via the Zn transport ZIP8 and/or by voltage-gated calcium channels [22], [23].

Methods used for analysis of Mn in tissues can be classified by detectable physical properties of Mn ions such as: i) mass; ii) atomic absorption; iii) an effect on the T_1_ relaxation time of protons and iv) X-ray emission. Inductively coupled plasma mass spectroscopy and atomic absorption spectroscopy are sensitive to low Mn concentrations, but cannot provide spatial information due to sample homogenization prior to measurement. Laser ablation inductively coupled plasma mass spectroscopy addresses this shortcoming and has measured Mn concentration with spatial resolutions down to 30 µm [24]–[27]. Secondary ion mass spectroscopy is an emerging technique which can provide spatial resolution of tens of nanometers [28], [29]. It remains to be demonstrated, however, whether this techniques can be used for Mn quantitation in brain tissues. Limitations may arise from the small penetration depth of primary ions, which results in a small sampling volume for mass spectroscopy analysis. Critical insights about the transport and distribution of Mn in the brain in the condition of Mn induced toxicity were obtained by MRI by measuring T_1_ relaxation time [30]–[32]. Although MRI can be performed *in vivo*, it cannot measure metal concentration and has limited spatial resolution. All remaining techniques use X-ray emission lines of Mn for imaging and quantitation. For instance, proton induced X-ray emission spectroscopy quantifies Mn in biological samples [33], but to our knowledge, no spatial maps of Mn distribution were obtained. Electron microscopy and electron energy-loss spectroscopy allow for imaging Mn at sub-cellular resolution, but sample treatment prior imaging alters the elemental distribution [34]. In summary, the aforementioned methods have disadvantages which limit their effectiveness at addressing Mn quantification and localization simultaneously.

X-Ray florescence (XRF) imaging serves as an alternative method with sensitivity and spatial resolution sufficient to reveal the distribution of Mn and other metals in cells and brain tissues [35]–[37]. This technique has unique capabilities for analyzing the distribution of metal ions in the context of metal induced neurotoxicity [38]. For XRF imaging, the X-ray beam can be focused to several microns to provide imaging of large areas such as coronal sections of rodent or human brain. Alternately, focusing on nanometer scale (currently down to 30 nm) allows for single cell imaging. By using XRF, we obtained high resolution maps of Mn distribution in the brains of rats chronically exposed to Mn, validated Mn quantitation by comparison with other established methods, and found that the substantia nigra compacta (SNc), the globus pallidus (GP) and the thalamus (Th) have the highest Mn content. The former structure is comprised primarily of dopaminergic neurons while the latter two contain GABAergic neurons. We also noted that the majority of Mn in the brain occurs in the mobile state and its location in the brain does not follow the pattern of Fe, Cu or Zn distributions.

## Materials and Methods

### Animals

Animal selection and treatment follow those described in [19] with modifications to diet (Purina rodent chow 5001, 70 ppm Mn content) and frequency of injections. Treated rats received intraperitoneal injections of 6 mg Mn/kg once daily on weekdays for four weeks while the control group received similar injections of saline solution. To prevent metal redistribution after animal sacrifice, we promptly froze dissected brains in liquid nitrogen. This study consists of two data collections using a total of 6 control and 7 treated animals. All experiments complied with animal rights regulations and were approved by the Institutional Committee on Animal Use at Purdue University.

### Preparation of brain sections

We sectioned brains using a cryotome fitted with a Teflon-coated blade. Four micron polypropylene film glued to plastic frames served as X-ray compatible sample supports. We selected Bregma sections displaying desired brain structures identified by cresyl violet staining. Coronal sections 10 µm and 30 µm thick were cut from frozen brains, placed on sample supports, frozen immediately, and stored at −80°C until analysis. To avoid modification of metal distribution and oxidation state, we did not apply chemical fixation.

### Immunohistochemistry

For immunohistochemistry (IHC), we used antibodies against the enzymes tyrosine hydroxylase (TH) and glutamic acid decarboxylase (GAD) as markers of dopaminergic and GABAergic neurons respectively, and Glial fibrillary acidic protein (GFAP) as a marker of astrocytes. Frozen (−80°C) brain sections on microscope slides or on 4 µm polypropylene film were thawed, fixed with 4% paraformaldehyde (PFA) in phosphate buffered saline (PBS; 137 mM sodium chloride, 2.7 mM potassium chloride, 10 mM disodium phosphate, and 1.8 mM monopotassium phosphate; pH 7.4) for 30 minutes at room temperature, washed twice with PBS, treated for 1 hour at room temperature sequentially with: 1) blocking/permeabilization solution: 2% bovine serum albumin (BSA)/0.2% (w/v) Triton X-100 in PBS), 2) primary antibodies in PBS containing 2% BSA: rabbit anti-GFAP at 1∶200 dilution or rabbit anti-TH at 1∶500 dilution, or rabbit anti-GAD65 and GAD67 at 1∶200, 3) secondary Alexa Fluor 488 goat anti-rabbit IgG antibodies in PBS containing 2% BSA at 1∶1000 dilution. Three, five minute washes with PBS were performed after treatment with primary and secondary antibodies. In a case of staining with anti-GAD65 and GAD67 heat-mediated retrieval of antigen was used. Rabbit serum was applied instead of primary antibodies for negative control. In addition to cell type identification, an antibody against the transferrin receptor (TfR) was used to compare the distribution of this carrier protein to that of Mn. IHC staining protocols are similar to those described above with changes to the primary antibody (anti-TfR at 1∶100 dilution applied for 2 hours) and to the secondary (Alexa Fluor 555 goat anti-mouse IgG). Mouse serum was accordingly used for negative control. After staining sections on microscope slides were subjected to ProLong Gold antifade reagent before being covered with a coverslip and sealed. Confocal images were acquired with Eclipse C1 Plus confocal system (Nikon Instruments Inc.), equipped with diode lasers and Eclipse TE2000-U inverted microscope. NIS-Elements BR 3.0 software was used to capture the images.

Selected sections on 4 µm polypropylene film were XRF imaged immediately after immunostaining and optical imaging. Treatments of brain tissue during staining result in significant loss of some metals compared to fresh, untreated sections, therefore data produced by IHC on adjacent sections was used for comparison with the XRF data from fresh-frozen samples.

Reagents used for IHC are as follows: anti-TH rabbit polyclonal (Abcam, ab6211); anti-GAD65 and GAD67 rabbit polyclonal (Abcam, ab49832); anti-GFAP rabbit polyclonal (Invitrogen, 18-0063); anti-TfR mouse monoclonal (Invitrogen, 13-6800); Alexa Fluor 488 goat anti-rabbit IgG, highly cross-adsorbed (Invitrogen, A11034); Alexa Fluor 555 goat anti-mouse IgG, highly cross-absorbed (Invitrogen, A21424); Rabbit serum (Sigma, R9133); BSA (Sigma, A4503); Triton X-100 (Sigma, T9284); ProLong Gold anti-fade reagent (Invitrogen, P36930).

### Synchrotron based XRF

Data collection was performed at the Advanced Photon Source Biophysics Collaborative Access Team facility [39]. For XRF imaging the beam profile was 7 µm×5 µm (h×v) and the pixel size was 40 µm×40 µm. At 10 keV, a flux of approximately 1.8×10^11^ photons/s was delivered to the sample. A pixel dwell time of 0.8 s was sufficient for proper counting statistics. Additionally, we placed two, 8 µm aluminum filters over the detector to attenuate the low Z element contributions to the spectrum, thereby avoiding detector saturation. We determined the detector location by maximizing the signal to noise ratio of the Mn Kα peak (unfitted) improving the signal∶noise by a factor of 15 as compared to measurements with no filters present. We used the MAPS program [40] to fit the collected data (Figure S1) on a per pixel basis. Thin film standards (NBS-1832/33, NIST) allow for metal quantification. Limits of detection and quantification we calculated as outline in [41] (Table S1). As an aside, we were able to obtain data on Mn distribution using a bending magnet beamline 10.3.2 of the Advanced Light Source with separate group of 4 treated and 4 control animals [42]. Data obtained at the 10.3.2 beamline had a systematic error due to the inability to record and fit detector spectrum and therefore are not included in this analysis. In spite of systematic error in absolute Mn quantitation, Mn was found to accumulate in same GP, Th, and SNc brain areas. The 10.3.2 beamline has since implemented detector spectrum recording at each pixel and, thus, is suitable for Mn imaging.

### Data Processing

**Figure 1:**
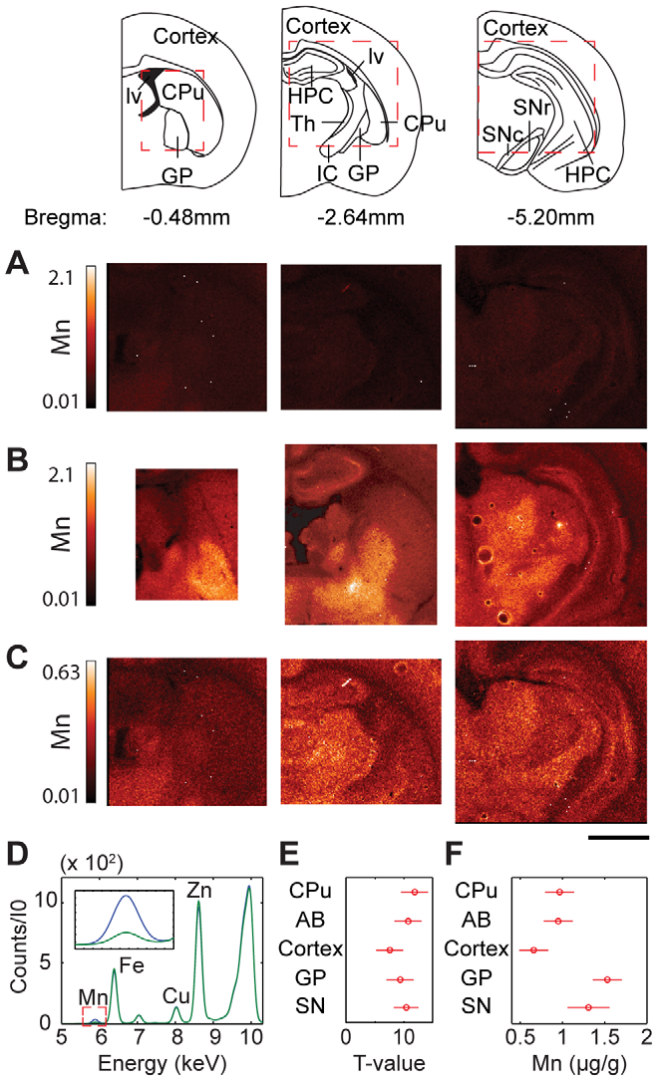
XRF imaging of Mn distribution in brain sections of control and treated rats. Mn distribution of coronal sections from untreated (control) rats (**A**) and Mn treated rats (**B**); from left to right; Bregma −0.48 mm, −2.64 mm, and −5.20 mm. Diagrams of these Bregma sections can be seen to the left where red dashed boxes roughly indicate the area(s) scanned. (**A**) and (**B**) demonstrate the increase of Mn concentration in treated samples. (**C**) The same image as (**A**) but with the scale adjusted in order to see contrast in the Mn distribution. All values given are in µg/g. Scale bar indicates a length of 2 mm. (**D**) Typical average pixel spectra for a control (green) and a treated sample (blue). Respective K_α_ peaks are labeled. The inset displays the Mn Kα peak. (**E**) T-test results show a statistically significant increase in Mn for treated samples as compared to control. Bars that cross a T-value of 0 indicate that the difference is not statistically significant. (**F**) ANOVA results of mean values of Mn concentration in treated samples for all areas studied. Overlapping bars indicate that the values are not significantly different. AB, axonal bundle; CPu, caudate putamen; GP, globus pallidus; HPC, hippocampal formation; IC, internal capsule; lv, lateral ventricle; Th, thalamus; SNc, substantia nigra compacta; SNr, substantia nigra reticular.

One sided T-tests determined the statistical significance of metal concentrations between the control and treated group (Figure 1E, Table 1), one-way analysis of variance (ANOVA) determined statistical significance of metal concentration between different brain regions within the treated group (Figure 1F) and an analysis of covariance determined the similarity of linear regressions. All statistical analysis used α = 0.05. Due to the inhomogeneous structure of the caudate putamen (CPu), we used k-means cluster analysis to objectively separate the ‘dark patches’ from the continuous field using the Zn signal (See Figure S2A). Contaminants or bubbles would result in a small cluster (<10 pixels) being identified; we deselected such pixels and repeated the analysis.

**Table 1:** Average concentrations for particular brain areas (mean ± SEM).

	Control (µg/g)				Treated (µg/g)			
Brain Area	Cu	Fe	Mn	Zn	Cu	Fe	Mn	Zn
AB	1.05±0.23	8.84±2.33	0.20±0.04	6.61±0.64	1.03±0.20	8.91±2.09	0.95±0.08*	6.40±0.41
Cortex	1.57±0.33	12.30±3.19	0.36±0.06	9.22±0.60	1.33±0.16	10.64±2.15	0.66±0.06*	8.67±0.71
CPu	1.45±0.18	11.23±1.16	0.22±0.04	8.72±0.45	1.58±0.14	11.66±0.85	0.97±0.08*	8.92±0.30
GP	1.28±0.26	14.20±1.85	0.40±0.05	7.91±0.58	1.13±0.15	13.01±1.29	1.53±0.10*	6.99±0.38*
Th	1.14	11.01	0.29	6.36	1.25±0.25	11.44±1.64	1.15±0.12*	6.54±0.48
SN	1.27±1.10	11.45±10.76	0.26±0.22	6.57±5.95	1.40±0.49	11.97±2.04	1.21±0.17*	6.46±0.51

* Denotes a significant difference from control (p<0.05).

AB, axon bundle; CPu, caudate putamen; GP, globus pallidus; SN, substantia nigra; Th, thalamus±.

## Results

### Animal model

Earlier human studies indicate that Mn-poisoned workers usually have blood Mn concentrations in the 4–15 µg/L range and a human study of 39 Mn-poisoned welders in Beijing revealed that the welders with distinct manganism had blood Mn levels between 8.2–36 µg/L [4]. The plasma Mn concentrations in treated animals at day 30 following the dose regimen were between 11–36 µg/L while Mn concentrations in untreated animals were between 3.5–5.6 µg/L [19], [21]. This model also demonstrates significantly increased Mn levels in brain tissue and in the cerebral spinal fluid as well as altered expression of divalent metal transporter 1 [43].

### Visualization of Mn distribution in the brain

The Mn distributions of untreated/control and treated coronal sections (Bregma −0.48 mm, −2.64 mm, and −5.20 mm) are displayed in Figure 1 and concentrations are given in Table 1. Mn concentrations measured by XRF are in good agreement with values reported in literature which used similar animal models and alternative measurement techniques (Table S2). Mn treatment results in a significant increase in Mn concentration in the brain as demonstrated by same scale comparison of Mn distributions in control and treated brains (Figure 1). Enhanced contrast is apparent for the Mn-treated brains with particular Mn enrichments in the GP and in the Th. When the Mn intensity scale is adjusted for the control samples, particular brain structures such as cortex and Th can similarly be visualized (Figures 1C and S2). T-test of untreated and Mn treated brains shows statistically significant increases in Mn concentration across all examined brain areas (Figure 1E, Table 1). Within the set of treated samples, ANOVA results show the statistically significant differences in Mn concentration between brain areas (Figure 1F).

Tissue treatment during IHC results in a considerable loss of Mn and a redistribution of Zn, while Fe and Cu are only partially affected (see Figure S3). Thus, tissue fixation or treatment prior to imaging would inevitably introduce an artificial effect on Mn content.

#### Choroid plexus

**Figure 2:**
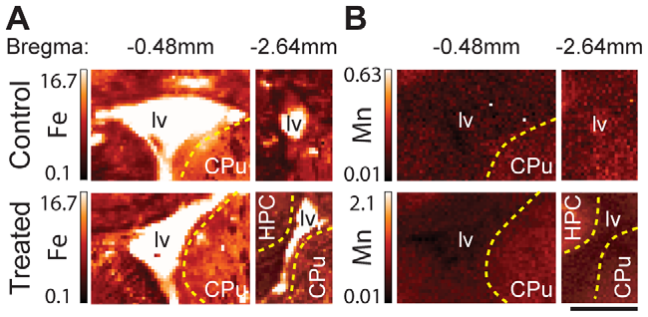
Mn and Fe distributions in the choroid plexus. XRF images of the Fe (**A**) and Mn (**B**) distributions in the choroid plexus (CP) within the lateral ventricle (lv) as identified by increased Fe signal. Images are Bregma −0.48 mm coronal sections of untreated rats (top) and Mn treated (bottom). Note that the images are not displayed on the same intensity scale, in order to see Mn contrast in the control image. Yellow dashed lines indicate the boundary between the ventricle and the labeled structures (CPu and HPC, hippocampal formation). The Fe signal shows the presence of CP (containing blood) within the ventricle. Mn concentration in the ventricle is lower than in adjacent brain structures of the CPu and HPC indicating clearance of Mn from the CP. All values given are in µg/g. Scale bar represents a length of 2 mm.

The CP consists of blood vessels and choroid epithelial cells. Blood within the CP produces a strong Fe signal assisting with the identification of the ventricle and CP (Figure 2A). Adjacent structures showed a weak Mn signal but no enhancement of the Mn signal was observed in the CP (Figure 2B). This result was observed for all coronal sections imaged. Our data demonstrate that 24 hours is a sufficient amount of time for the body to remove Mn content from the CP. This result is in agreement with previous publications using radiography [44] and MRI [31], [45] showing Mn in CP within 10 min post injection and its clearance 24 hours later.

#### Caudate putamen

**Figure 3:**
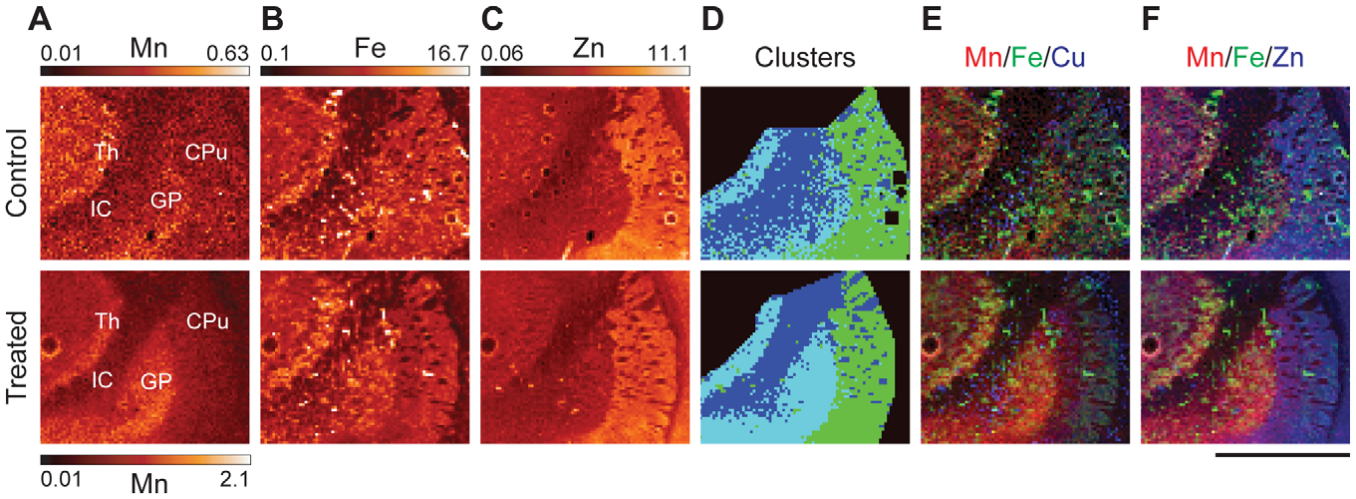
XRF imaging of the caudate putamen, globus pallidus, thalamus, and internal capsule. (**A–C**) Mn, Fe, and Zn XRF images in control and treated rats. Maximum intensity for the control Mn images is 30% of the maximum for the treated images. All numbers are given in µg/g. (**D**) Image displaying the results of cluster analysis. (**E & F**) Tri-colored plots displaying Mn, Fe, & Cu/Zn as green, red, and blue respectively. Scale bar represents a length of 2 mm.

The CPu is the structure which provides inhibitory input to the GP. In turn, it receives excitatory inputs from the SNc via dopamine and from the cortex via glutamate. The CPu is enriched with Zn in both untreated and treated samples, while the GP is enriched with Mn in Mn intoxicated brains. The border between the CPu and GP can be readily identified in both groups by examining the decrease in Zn concentration or in treated samples by studying the increase in Mn concentration (Figures 3A, 3C, and S4). Distribution of Fe, Cu, and Zn in the CPu is not uniform with large patches of low concentration of all three metals. These patches are axon bundles (AB) that permeate the CPu from the internal capsule (Figure 3). Cluster analysis using the Zn signal separated Zn and Fe enriched areas which form the continuous field (labeled CPu) from axon bundles. Although the CPu is enriched with Cu, Fe and Zn as compared to the axons, both structures accumulated the same Mn content in treated brains (Figure 3A, Table 1). This conveys that Mn absorption and retention in these two types of brain tissue appear to be independent of the content of other three metals.

#### Globus Pallidus and Thalamus

The GP mostly contains GABAergic neurons which signal to the Th. It receives inhibitory input from the CPu and excitory glutamate input from the subthalamic nuclei. The GP is the largest continuous area of the brain which accumulates the highest Mn content, (see Figures 1, 3, and S2). Interestingly, Mn accumulation in the GP resulted in a statistically significant decrease of Zn content in this region (Table 1). As human autopsies have revealed neurodegeneration in the GP [6], we checked for GAD positive neurons and possible astrogliosis in this area (Figure S5). GAD and GFAP immunostained sections of treated and control brains do not demonstrate a significant difference in the content of GAD positive neurons or astrocytes between sample groups (Figure S5). As astrocyte content was determined to be the same, distinct Mn accumulation in GP is not due to Mn accumulation in astrocytes. In other brain areas enriched with astrocytes such as subventricular zone, the distribution of astrocytes, visualized by GFAP, did not correlate with distribution of Mn.

#### Substantia nigra (SN)

**Figure 4:**
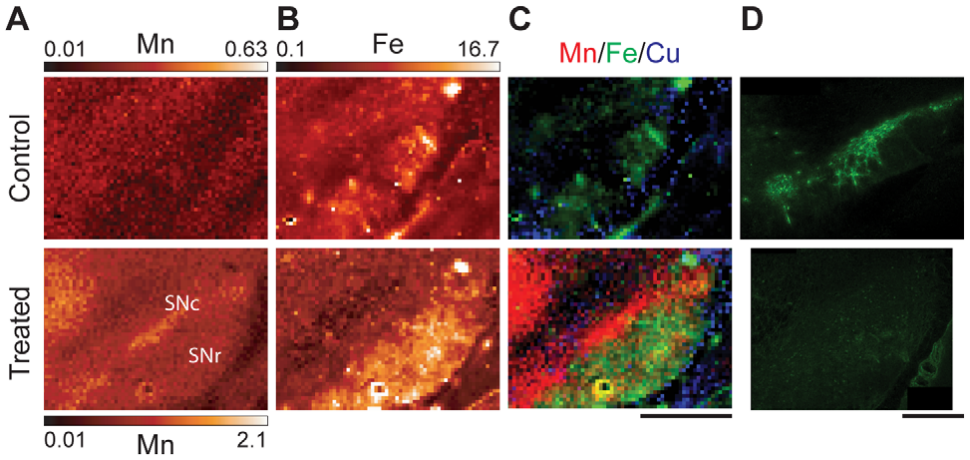
XRF imaging of the substantia nigra of control and treated samples. XRF images of Mn (**A**) and Fe (**B**) for treated and control samples. Note that the maximum Mn intensity for the control sample is 30% of the treated maximum intensity. Numbers given are in µg/g. (**C**) Tri-colored image of the SN where red, green, and blue represent Mn, Fe, and Cu respectively (same scale for Mn). Scale bar represents a length of 1 mm. (**D**) Confocal images of tyrosine hydroxylase stained SN area of adjunct sections recorded in identical experimental conditions.

The SNc is a relatively thin layer (300–500 µm) containing densely packed cell bodies of dopaminergic neurons which provide input to the CPu. The SNc is located dorsal to the larger and more diffuse SN pars reticular (SNr), comprised primarily of GABAergic neurons. Mn treatment results in an increased Mn signal in the SNc (Figure 4A). Area of increased Mn content contrasts the dorsal side of the SNr which can be readily identified by its position and increased Fe signal (Figure 4B). Dopaminergic neurons of the SNc were identified by TH immunostaining (Figures 4D and S5). However, Mn intoxication consistently resulted in a decrease in the intensity of TH stain in the SNc (Figure 4D), which is in agreement with a previous study [46]. Dopaminergic processes also occur in the CPu, however TH staining there was determined to be weak and cannot be reliably distinguished from background due to unspecific staining (Figure S5). Comparison of the TH stain and Mn XRF signal show that the SNc is enriched with dopaminergic neurons and accumulates Mn.

#### Cortex and Hippocampus

Mn has uniform distribution in the cortex and its concentration is lowest here in comparison with other analyzed brain areas. The hippocampus has some areas (dentate gyrus and CA3 of Ammon's horn) with increased Mn accumulations (Figures 1 and S2), As this structure is not a part of the basal ganglia system, detailed analysis of metal distribution in this brain area will be presented elsewhere.

### Interplay between the Fe, Cu, Zn, and Mn distributions

Visual inspection of images shows the absence of correlation as areas of highest Fe, Cu or Zn content do not demonstrate proportionally high Mn. To quantify this result, we analyzed scatter plots of individual pixel data for each brain structure separately (Figures S6 and Table S3). This level of resolution with 40 µm×40 µm pixel size probed by 7 µm×5 µm X-ray beam is close to monitoring single cells. Associated linear regression fits and statistics calculated for each of the areas examined in this study are displayed in Figure S6 and Table S3. We observed moderate correlations in the cortex (Fe/Mn), in the GP (Fe/Mn and Zn/Mn) and in the SN (Fe/Mn, Cu/Mn, and Zn/Mn) of control samples. In treated samples, we observed moderate Zn/Mn correlations in the cortex and the SN. The majority of scatter plots demonstrate a change in the linear regression slopes between control and treated samples.

**Figure 5:**
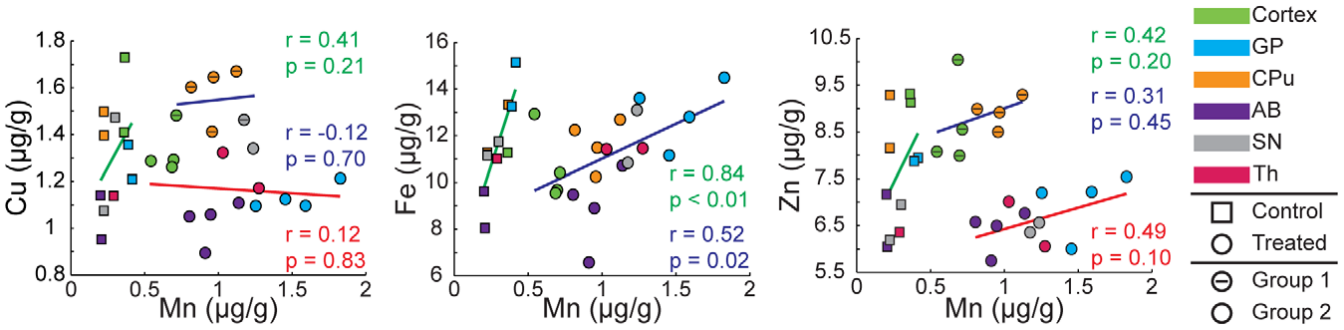
Scatter plots and correlations for control and treated groups. Data points calculated by taking the average of the brain areas of interest for control and treated samples. We performed cluster analysis (2–5 clusters) on the data obtained from treated samples to objectively identify similar data points in terms of metal concentrations. Clustering was done using Cu/Mn, Fe/Mn, or Zn/Mn at equal weights. We found that there was no clear grouping according to Fe/Mn while according to Cu/Mn or Zn/Mn two clusters were appropriate (Group1 and Group2). For Zn/Mn, cluster analysis resulted in two exclusive groups in terms of brain structures. Color matched Pearson's correlation coefficients and *p-values* are also given. Linear regression parameters and statistical analysis results are given in Table S4.

Metal content was also integrated over a selected brain structure on individual brain sections and presented in scatter plots (Figure 5). A correlation displayed in a scatter plot can indicate transport and/or accumulation by a common mechanism. There is a strong Fe/Mn correlation for both controlled and treated brains with *r = 0.84*, *p<0.01* and *r = 0.52*, *p = 0.02* respectively. IHC staining for TfR did not demonstrate a significant difference between control and treated groups for a given brain region; higher expression was observed in the GP and SNc while the CPu and cortex had lower levels of expression (Figure S7). This trend in TfR distribution qualitatively follows the trend in Mn distribution, suggesting that Mn, like Fe, may use the transferrin-TfR pathway to gain entry into the cell [47], [48]. To objectively identify data points similar in terms of metal concentration, cluster analysis was performed (2–5 clusters) on the set of data obtained from treated sections (*n = 20*) using Cu/Mn, Fe/Mn, or Zn/Mn at equal weights. The results of this analysis determined that there was no clear grouping according to Fe/Mn and therefore the data was treated as a single set, while according to Cu/Mn or Zn/Mn, two clusters were appropriate. Clustering according to Cu/Mn did not result in the categorization of brain structures into mutually exclusive groups; one group consisted of the CPu data points and one data point from each the SN and the cortex (*n = 6*) with the remaining points in the second group (*n = 14*). Conversely, Zn/Mn clustering resulted in two disjoint groups; one group consisting of the data from the CPu and cortex (*n = 8*) and the second group consisting of data from the GP, AB, SN and Th (*n = 12*). We observed moderate correlations for Zn/Mn in control and both treated groups; *r = 0.42*, *r = 0.31*, and *r = 0.49* respectively, however more data points are necessary for sufficient statistical power (*p = 0.20*, *p = 0.45*, & *p = 0.10*). These data suggest possibilities of a shared transport and/or sequestration mechanism of Fe/Mn, and to a lesser extent of Zn/Mn.

## Discussion

Exposure of the general population to Mn commonly comes from environmental pollution and dietary intake while sub-populations are at an increased risk of manganism due to occupational exposure, drug use, or compromised liver function. Environmentally, the gasoline additive methylcyclopentadienyl Mn tricarbonyl can increase Mn emissions in air [49]. Dietary sources of Mn are fruits, vegetables, grains, and nuts with a 2–5 mg/day intake deemed safe and adequate [50]. Mn naturally occurs within ores, thus manganism is prevalent within the mining, smelting, and welding industries [4], [7]–[15]. Chronic users of methcathinone (or ephedrone), a controlled substance synthesized using potassium permanganate, also demonstrate symptoms of manganism [51]. Patients suffering from hepatic failure have been reported to have increased Mn blood levels [52]. As Mn effects are mostly neurotoxic, analysis of Mn distribution in brain is critical for understanding effects of Mn overexposure.

XRF imaging provided new insight on the effects of chronic Mn exposure in the rat model of neurobiochemical defects mimicking those observed in humans. Statistically significant increases in Mn content were observed in the AB, cortex, and basal ganglia with particularly high Mn concentrations in areas enriched with GABAergic neurons (GP & Th) and dopaminergic neurons (SNc) (Figures 1, 3, 4, and Table 1). The subventricular zone, an area composed primarily of astrocytes, did not display increased Mn signals in comparison to the nearby CPu (Figure 2) ruling out the possibility of preferential Mn accumulation in astrocytes.

While Mn accumulation in the GP is well established, Mn detection in the SNc is a novel result achieved due to increased spatial resolution of the detection technique. Previous studies have observed Mn in the SN as detected by MRI in monkeys exposed to Mn [53], in patients with liver failure [52], and in ephedrone users [54]. Conversely, post-mortem examination of the SN has revealed no noticeable effect on pigmented cells of the human brain [3]. Concentration of Mn detected in the SNc and GP here by XRF is about 40 µM, which is less than the 100 µM concentration determined to be toxic for cells in cell cultures [55]. Currently, we cannot exclude that higher resolution (0.3 µm×0.3 µm) imaging might uncover areas of local Mn concentration greater than 40 µM. However, effects of prolonged exposure to sub-toxic levels of Mn ions still can be considerable. Here, Mn intoxication results in a decrease in TH stain in the SNc similar to that previously reported [46] (Figure 4D). Sensitivity of dopaminergic cells to Mn has been noted before; for instance in several cell culture models Mn has been shown to interact with cellular dopamine by disrupting mitochondrial respiration and inhibiting the antioxidant system thereby straining the cell's ability to combat oxidative stress [36], [56], [57]. Alternatively, Mn potentially serves as a catalyst in the autoxidation of dopamine [58], [59] and has an increased toxicity when accompanied by dopamine or L-DOPA treatments [60], [61]. A rodent model of pre-parkinsonism has shown a high sensitivity to Mn toxicity and exhibited significant neurobehavioral impairments when Mn treatment is followed by unilateral lesion with 6-hydroxydopamine [62]. As this analysis was done with rodent model, selective accumulation of Mn in the SNc remains to be demonstrated for human brains.

Analyzes of Mn distribution in samples subjected to immunochemical treatment prior to XRF imaging demonstrated that Mn can be easily lost by such treatments (Figure S3). Thus, we conclude that majority of detected Mn is present in relatively mobile form. Due to this result, analysis of Mn distribution in fresh frozen tissue (as reported here) and in cell culture [36] will be different from these obtained involving intense tissue processing, such as cell fractionation [63] and chemical treatment of tissue [34]. Detection of largely labile form of accumulated Mn means that chelation therapy should be effective in removing excess Mn burden from the brain. To date, chelation therapy in exposed individuals remains the primary treatment for manganism [21], [64], [65].

Mechanisms resulting in selective accumulation of Mn in the GP, Th, and SNc are currently unknown. Intriguingly the border between CPu and GP can be easily visualized in treated samples using the Mn distribution, even though both structures have GABAergic neurons. We demonstrate that Mn accumulation in the brain targets particular groups of neurons rather than follows the distribution of any other biologically relevant ion. We do not explicitly discuss Ca^2+^ due to its highly homogeneous distribution in the brain or Mg^2+^ due to the strong attenuation of the signal by the aluminum foils (Figure S1). Biochemical properties of Mn accumulating neurons including their metabolic activities, excitability [66], [67], presence of Mn binding molecules, and slow clearance of Mn are likely responsible for detected Mn accumulation but more studies are needed to delineate these multiple potential contributions. XRF technique is a perfect tool for analysis of mechanisms of Mn transport and accumulation in the brain. For instance, various proteins suspected to play the role in these processes can be selectively knock out in cell or animal models and changes in the Mn distribution can be visualized by XRF.

When accumulated in the neurons, Mn can have neurotoxic action by various mechanisms including Mn effect on mitochondria [68]–[70] as well as alteration of other essential metal ions [19]. For example, under Mn intoxication, an intracellular decrease of Fe for cells in culture [36] and an increase in Fe content in particular brains areas [21] were reported. Here we determined strong correlation for Fe/Mn (Figure 5) indicating possible transport and/or sequestration of these two ions by the same mechanism. We found no statistically significant changes in Fe or Cu content as a result of Mn intoxication (Table 1) but statistically significant decrease in Zn in GP. In PD elevated Fe content in SN is established [35]. It could be that elevated Fe can be detrimental to cell survival in the similar way as elevated Mn, via initiating oxidative stress and dopamine oxidation. Single cell resolution XRF imaging of the dopaminergic neurons will be use in the future to analyze Mn/Fe distributions in these cells at different progression of Mn toxicity.

## Conclusions

We present for the first time *in situ* quantification and distribution of Mn in Mn-exposed rodents obtained using synchrotron x-ray fluorescence. In agreement with other established methods, Mn concentration was observed to be the highest within structures of the basal ganglia, specifically the GP, Th and SNc. XRF allowed us to monitor content of other biologically relevant metal ions (such as Fe, Cu, Zn) and correlate their distribution with distribution of Mn. We demonstrated that Mn distribution does not co-localize with these of Fe, Cu or Zn. However, the correlation in the Fe/Mn couple was observed in between as well as within different brain structures suggesting the common transport mechanism.Zn level in the GP was statistically lower in Mn treated animals, possibly indicating a competition for a transport mechanism into the cells of this region. Alternatively, the decreased Zn content could be the result of a competition for a sequestration mechanism within the cells of the GP. Unlike previous studies which either lack sufficient resolution or require sample fixation/treatment, we observed a high concentration of Mn localized in the SNc which is primarily composed of dopaminergic neurons. In addition to accumulation in the GP, Mn accumulation and toxic action in this region could account for Parkinsonism observed for Mn intoxication.

## Supporting Information

Figure S1 **Spectra and fitting results.** Representative spectrum (black) from control sample (**A**) and Mn treated sample (**B**) with corresponding fit (red) and background (blue) obtained using the MAPS program [40]. The Kα peaks of the metals of interest have been labeled accordingly. The presence of the Al foil over the detector results in suppressed peaks at the lower energies and subsequently a less precise fit of the data.
(TIF)

Figure S2 **XRF images of metal distribution in control and treated samples.** (**A**) Results of cluster analysis performed on the caudate putamen. (**B**) Images of metal distribution. All numbers are in µg/g. Scale bar represents a length of 2 mm.
(TIF)

Figure S3 **Immunohistochemical staining effect on metal distribution.** (**A**) XRF images of a treated sample after tyrosine hydroxylase staining. Note that the Zn distribution in the hippocampal formation (HPC) has been drastically altered in the staining process as compared to unstained, treated sample (**B**) and unstained, control sample (**C**). Similarly, Mn has been washed from the HPC, which generally is identifiable using Mn signal. The Mn intensity scale has been adjusted for the control sample to be 30% of the maximum intensity of the treated samples. Despite washing, the dentate gyrus and CA1 of Ammon's horn are still visible using the Fe signal, however other areas have decreased intensity. Similarly, as apparent by the lack of a strong Fe signal to the lower right of the HPC in (**A**), the choroid plexus has also been washed. Cu looks to be strongly bound along the ventricle wall, but has otherwise been redistributed. All values given are in µg/g. Scale bar represents a length of 2 mm.
(TIF)

Figure S4 **CPu/GP boundary.** Mn and Zn XRF images of (**A**) control and (**B**) Mn treated samples. Given numbers are in µg/g. Scale bar represents a length of 1 mm. A normalized intensity profile along for both samples is given below the XRF images. For both samples we observe an increase in Mn at the caudate putamen (CPu)/globus pallidus (GP) boundary (solid blue line), which is accompanied by a decrease in Zn content (dashed red line). A five pixel (200 micron) line width was used to obtain the intensity profile and is indicated on the XRF images by the black arrow. The GP/axonal bundle (AB) boundary in the control section is only approximate whereas in the treated section the decrease in Mn concentration is easily seen.
(TIF)

Figure S5 **GAD, GFAP, & TH immunostaining.** From left to right; Control, Mn treated, and negative control. (**A**) Glutamic acid decarboxylase (GAD) and (**B**) glial fibrillary acidic protein (GFAP) immunostaining of the globus pallidus (GP). (**C**) Tyrosine hydroxylase (TH) immunostaining of caudate putamen (CPu). Negative control shows unspecific binding of secondary antibodies in CPu in control brain section. (**D**) TH immunostaining of the substantia nigra (SN). Confocal images in any given row were taken at 10× magnification under the same microscope settings.
(TIF)

Figure S6 **Pixel scatter plots.** CPu, caudate putamen; GP, globus pallidus; SN, substantia nigra.
(TIF)

Figure S7 **Transferrin receptor immunostaining.** Transferrin immunostaining of control (top) and Mn treated (bottom) sections. Negative control shows unspecific binding of secondary antibodies in SN in a control brain section. All images, unless indicated otherwise, were taken at 10× magnification under the same microscope settings. CPu, caudate putamen; GP, globus pallidus; IC, internal capsule; SNc, substantia nigra compacta; SNr, substantia nigra reticular; Th, Thalamus.
(TIF)

Table S1 **Minimum detection limits and minimum analyzable limits.** Spectra were taken of NIST standards (formerly NBS 1832/1833) which were used to determine the minimum detection limits (MDL) and minimum analyzable limits (MAL). For the MDL calculation, a linear background was approximated as linear and a 95% confidence threshold was used, corresponding to a signal of 1.654σ above the background. For the MAL calculation, standard practice is to deem a peak sufficient for quantification if it is 10 sigma above the background, i.e. σ/peak = 0.1 (α = 0.1). Note that values are for exact conditions of reported XRF experiment and not for XRF in general as XRF measurements can be performed with significantly different parameters. All values reported in Table 1 exceed the MAL by more than an order of magnitude with the exception of Mn in the control sample, which is 2–4 times larger than the reported MAL.
(DOCX)

Table S2 **Manganese concentrations reported in literature.**
(DOCX)

Table S3 **Linear regression parameters for pixel scatter plots.**
(DOCX)

Table S4 **Linear regression parameters for brain region scatter plot.**
(DOCX)
