# Supplementary figures and images for: X-Ray Fluorescence Imaging: A New Tool for Studying Manganese Neurotoxicity
Source: PLoS One. 2012 Nov 19;7(11):e48899. doi: 10.1371/journal.pone.0048899 (PMC3501493; doi:10.1371/journal.pone.0048899)

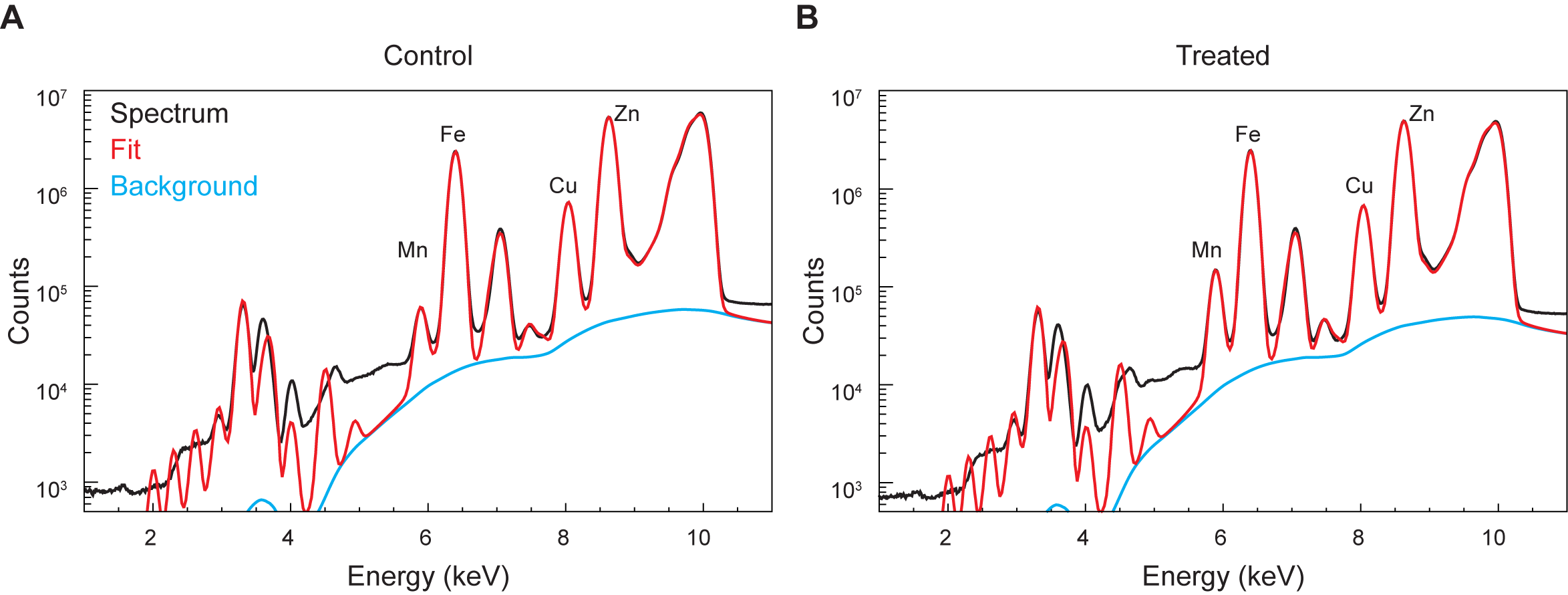

Supplement: Figure S1 — Spectra and fitting results. Representative spectrum (black) from control sample (A) and Mn treated sample (B) with corresponding fit (red) and background (blue) obtained using the MAPS program [40]. The Kα peaks of the metals of interest have been labeled accordingly. The presence of the Al foil over the detector results in suppressed peaks at the lower energies and subsequently a less precise fit of the data. (TIF) [file pone.0048899.s001.tif]

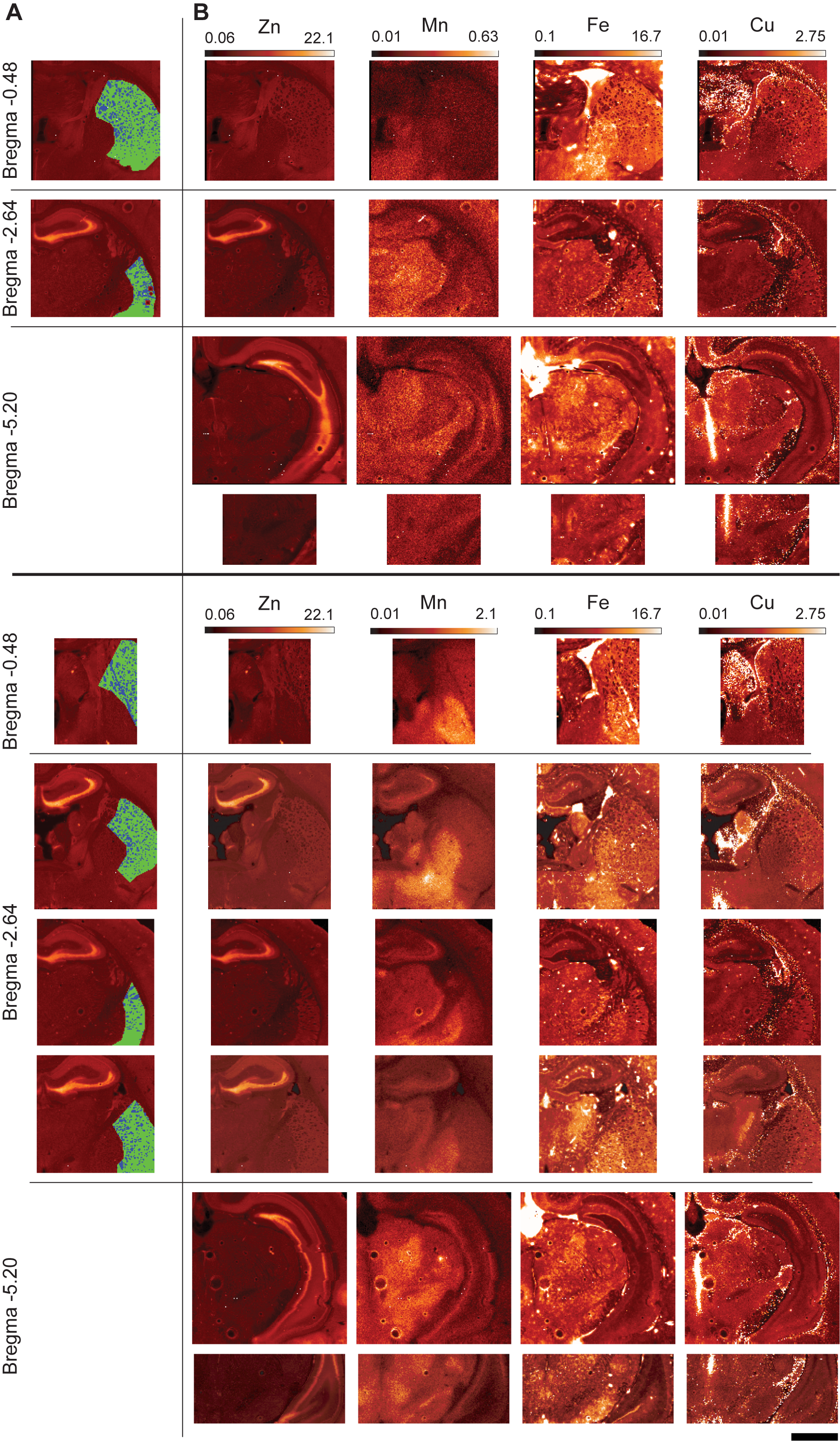

Supplement: Figure S2 — XRF images of metal distribution in control and treated samples. (A) Results of cluster analysis performed on the caudate putamen. (B) Images of metal distribution. All numbers are in µg/g. Scale bar represents a length of 2 mm. (TIF) [file pone.0048899.s002.tif]

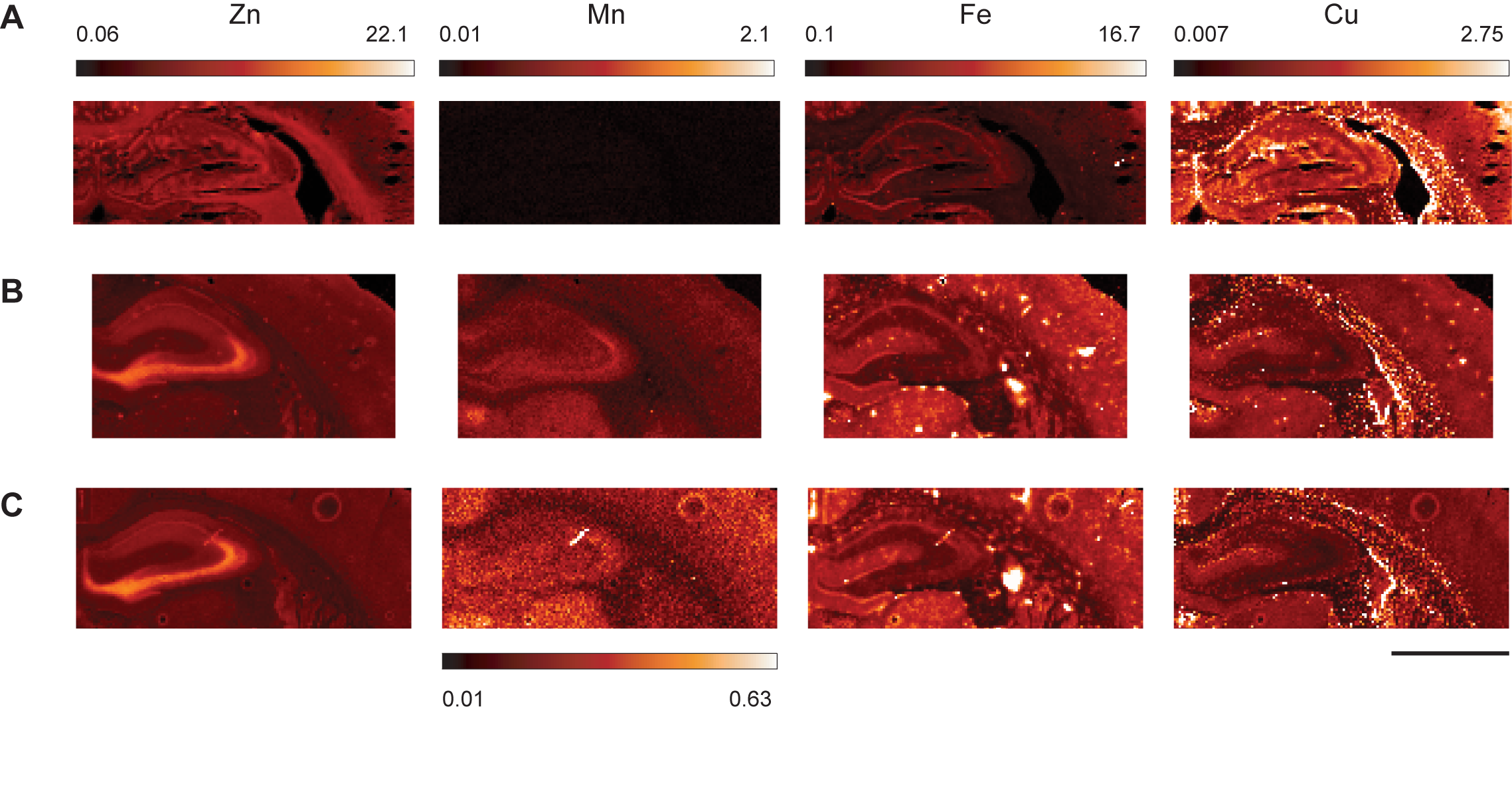

Supplement: Figure S3 — Immunohistochemical staining effect on metal distribution. (A) XRF images of a treated sample after tyrosine hydroxylase staining. Note that the Zn distribution in the hippocampal formation (HPC) has been drastically altered in the staining process as compared to unstained, treated sample (B) and unstained, control sample (C). Similarly, Mn has been washed from the HPC, which generally is identifiable using Mn signal. The Mn intensity scale has been adjusted for the control sample to be 30% of the maximum intensity of the treated samples. Despite washing, the dentate gyrus and CA1 of Ammon's horn are still visible using the Fe signal, however other areas have decreased intensity. Similarly, as apparent by the lack of a strong Fe signal to the lower right of the HPC in (A), the choroid plexus has also been washed. Cu looks to be strongly bound along the ventricle wall, but has otherwise been redistributed. All values given are in µg/g. Scale bar represents a length of 2 mm. (TIF) [file pone.0048899.s003.tif]

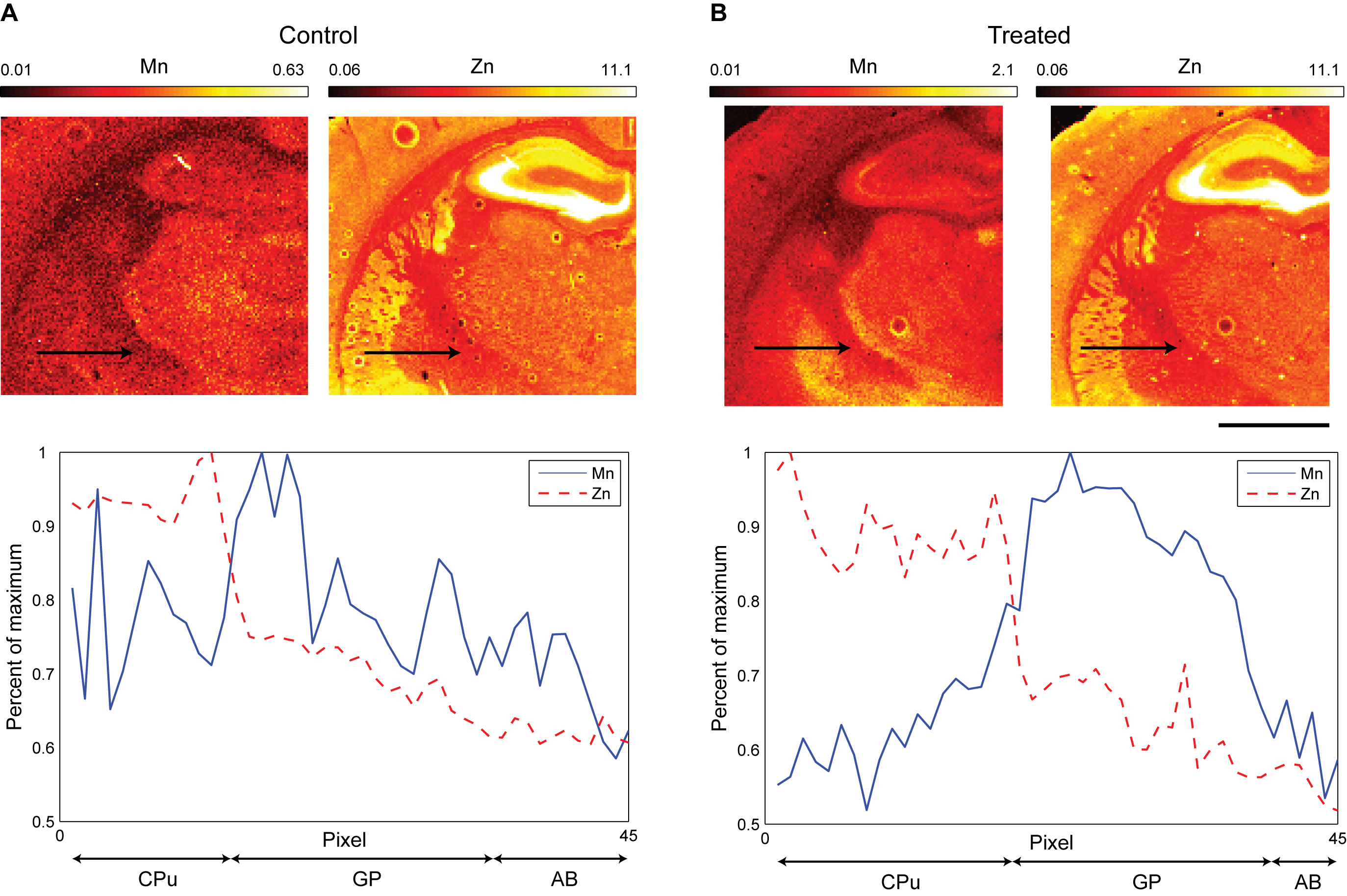

Supplement: Figure S4 — CPu/GP boundary. Mn and Zn XRF images of (A) control and (B) Mn treated samples. Given numbers are in µg/g. Scale bar represents a length of 1 mm. A normalized intensity profile along for both samples is given below the XRF images. For both samples we observe an increase in Mn at the caudate putamen (CPu)/globus pallidus (GP) boundary (solid blue line), which is accompanied by a decrease in Zn content (dashed red line). A five pixel (200 micron) line width was used to obtain the intensity profile and is indicated on the XRF images by the black arrow. The GP/axonal bundle (AB) boundary in the control section is only approximate whereas in the treated section the decrease in Mn concentration is easily seen. (TIF) [file pone.0048899.s004.tif]

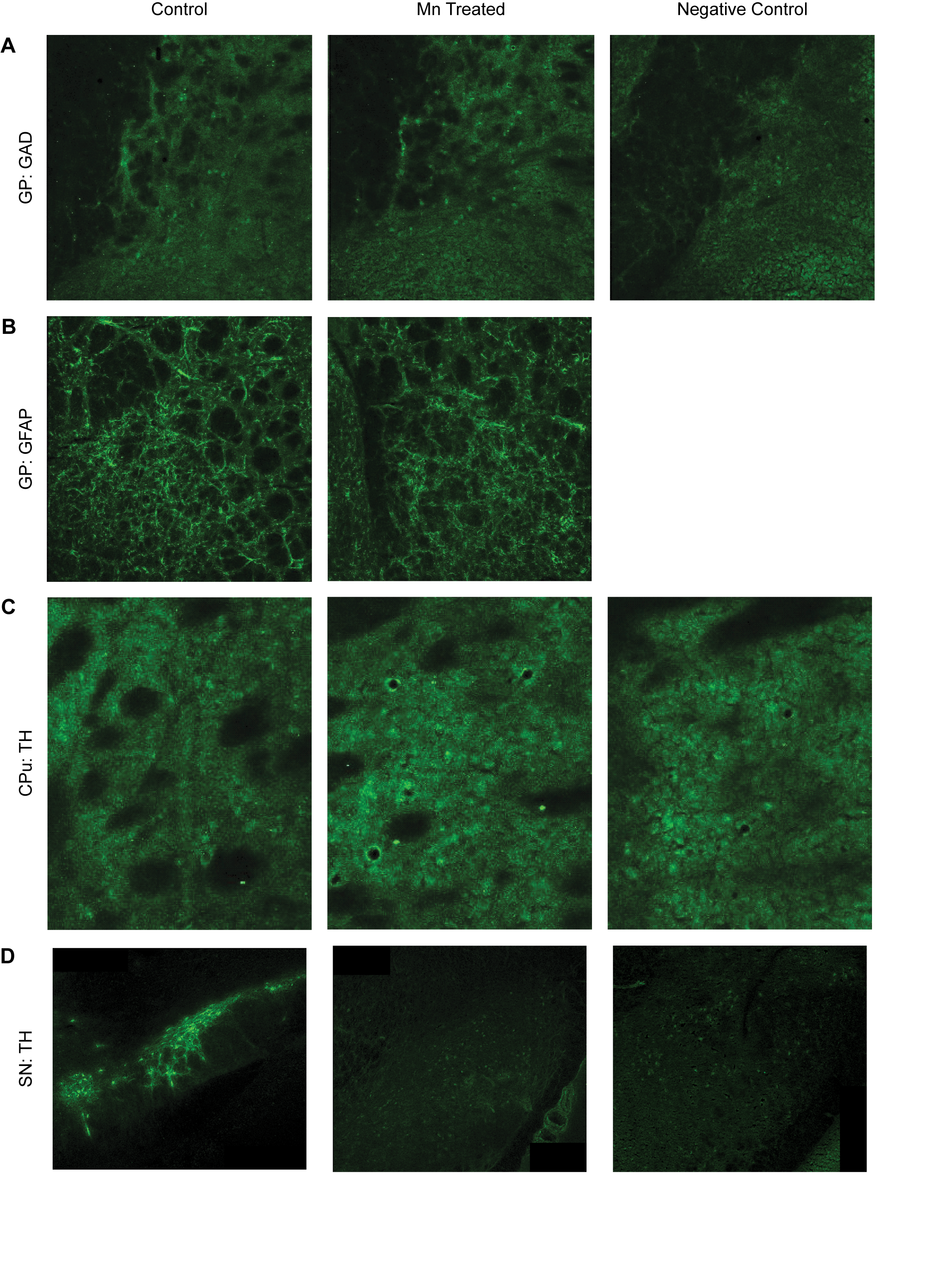

Supplement: Figure S5 — GAD, GFAP, & TH immunostaining. From left to right; Control, Mn treated, and negative control. (A) Glutamic acid decarboxylase (GAD) and (B) glial fibrillary acidic protein (GFAP) immunostaining of the globus pallidus (GP). (C) Tyrosine hydroxylase (TH) immunostaining of caudate putamen (CPu). Negative control shows unspecific binding of secondary antibodies in CPu in control brain section. (D) TH immunostaining of the substantia nigra (SN). Confocal images in any given row were taken at 10× magnification under the same microscope settings. (TIF) [file pone.0048899.s005.tif]

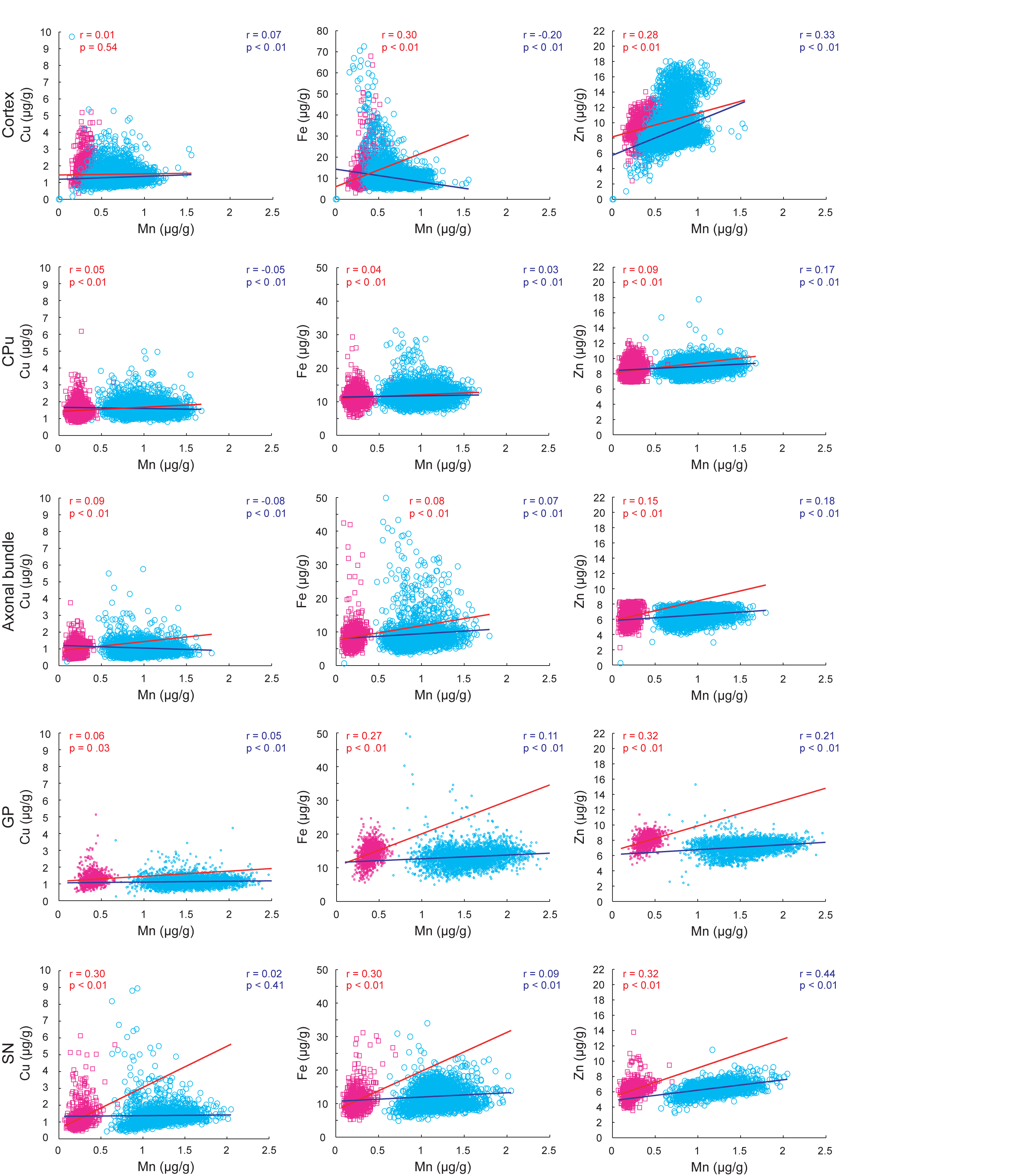

Supplement: Figure S6 — Pixel scatter plots. CPu, caudate putamen; GP, globus pallidus; SN, substantia nigra. (TIF) [file pone.0048899.s006.tif]

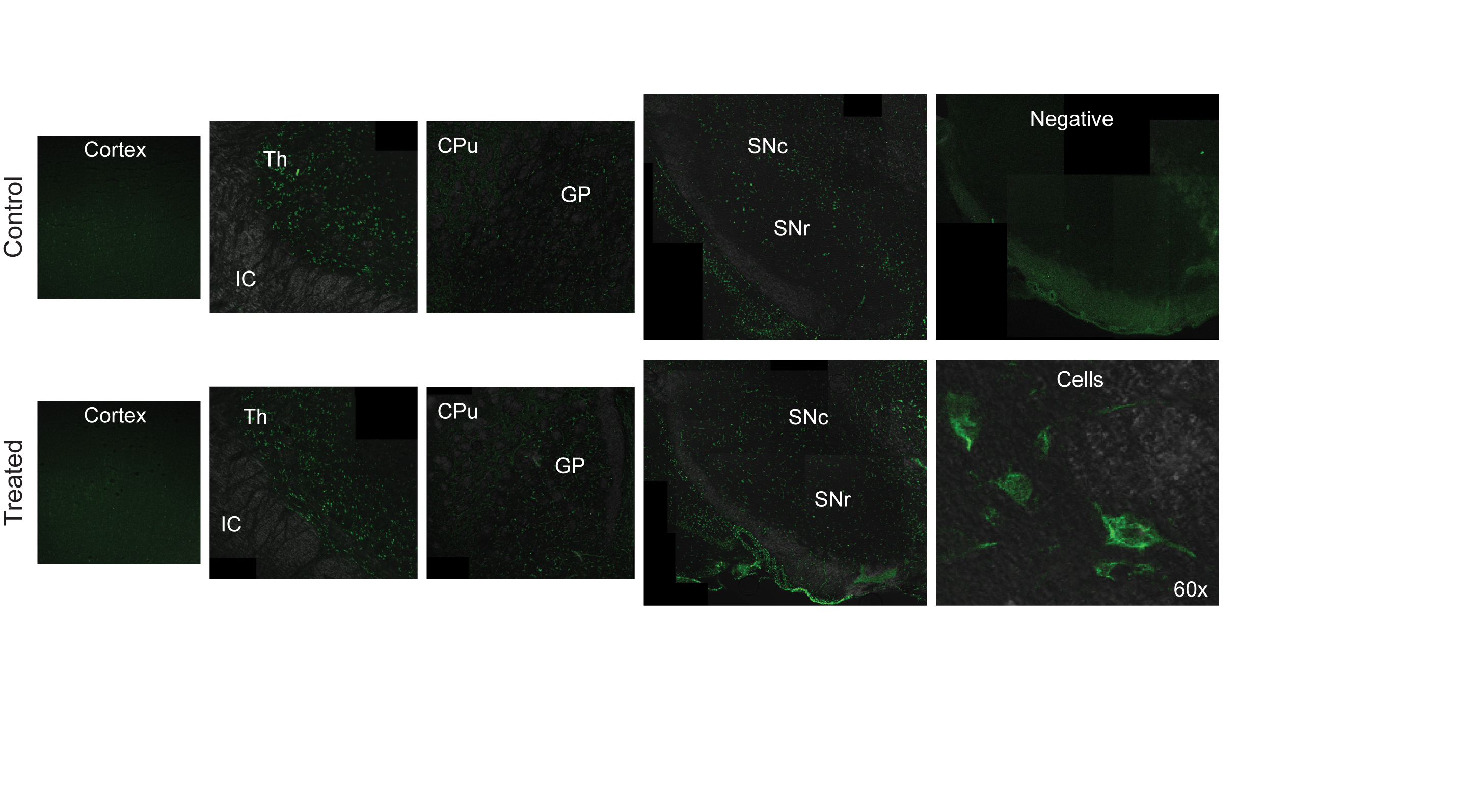

Supplement: Figure S7 — Transferrin receptor immunostaining. Transferrin immunostaining of control (top) and Mn treated (bottom) sections. Negative control shows unspecific binding of secondary antibodies in SN in a control brain section. All images, unless indicated otherwise, were taken at 10× magnification under the same microscope settings. CPu, caudate putamen; GP, globus pallidus; IC, internal capsule; SNc, substantia nigra compacta; SNr, substantia nigra reticular; Th, Thalamus. (TIF) [file pone.0048899.s007.tif]
