# Supplementary material for: X-Ray Fluorescence Imaging: A New Tool for Studying Manganese Neurotoxicity
Source: PLoS One. 2012 Nov 19;7(11):e48899. doi: 10.1371/journal.pone.0048899 (PMC3501493; doi:10.1371/journal.pone.0048899)
Supplement: Table S1 — Minimum detection limits and minimum analyzable limits. Spectra were taken of NIST standards (formerly NBS 1832/1833) which were used to determine the minimum detection limits (MDL) and minimum analyzable limits (MAL). For the MDL calculation, a linear background was approximated as linear and a 95% confidence threshold was used, corresponding to a signal of 1.654σ above the background. For the MAL calculation, standard practice is to deem a peak sufficient for quantification if it is 10 sigma above the background, i.e. σ/peak = 0.1 (α = 0.1). Note that values are for exact conditions of reported XRF experiment and not for XRF in general as XRF measurements can be performed with significantly different parameters. All values reported in Table 1 exceed the MAL by more than an order of magnitude with the exception of Mn in the control sample, which is 2–4 times larger than the reported MAL. (DOCX) [file pone.0048899.s008.docx]

**Table S1. Minimum detection limits and minimum analyzable limits**

| Element | C_std_ (μg/cm^2^) | Total (counts) | Background (counts) | Signal (counts) | C_MDL_ (μg/g) | C_MAL_ (μg/g) |
| --- | --- | --- | --- | --- | --- | --- |
| Mn | 4.22 | 3.45E+05 | 3.02E+03 | 3.42E+05 | 1.67E-02 | 1.01E-01 |
| Fe | 14.15 | 1.71E+05 | 7.74E+02 | 1.70E+05 | 5.67E-02 | 3.46E-01 |
| Cu | 2.25 | 6.02E+05 | 1.33E+04 | 5.89E+05 | 1.08E-02 | 6.57E-02 |
| Zn | 3.98 | 1.44E+05 | 1.07E+03 | 1.43E+05 | 2.23E-02 | 1.36E-01 |
