# Supplementary material for: X-Ray Fluorescence Imaging: A New Tool for Studying Manganese Neurotoxicity
Source: PLoS One. 2012 Nov 19;7(11):e48899. doi: 10.1371/journal.pone.0048899 (PMC3501493; doi:10.1371/journal.pone.0048899)
Supplement: Table S2 — Manganese concentrations reported in literature. (DOCX) [file pone.0048899.s009.docx]

**Table S2. Manganese concentrations reported in literature**

| Source | Method | Control (μg/g) | Treated (μg/g) |
| --- | --- | --- | --- |
| Cortex | | | |
| APS | XRF | 0.36 ± 0.06 | 0.66 ± 0.06 |
| [[1](#_ENREF_1)] | AAS | 0.43 ± 0.03 | 0.79 ± 0.04 |
| [[2](#_ENREF_2)] | AAS | 0.99 ± 0.27 | 0.99 ± 0.33 |
| [[3](#_ENREF_3)] | ICP-MS | 0.97 ± 0.26 | 3.07 ± 1.02 |
| [[4](#_ENREF_4)] | PIXE | 1.6 | 3.2 ± 0.6 |
| [[5](#_ENREF_5)] | INAA | 0.34 ± 0.07 | 0.83 ± 0.16 |
| [[6](#_ENREF_6)] | INAA | .263 ±0.044 | 0.311 ±0.060 |
| [[7](#_ENREF_7)] | LA-ICP-MS | 0.20 ± 0.03 |  |
| CPu | | | |
| APS | XRF | 0.22 ± 0.04 | 0.97 ± 0.08 |
| [[1](#_ENREF_1)] | AAS | 0.42 ± 0.05 | 0.94 ± 0.20 |
| [[2](#_ENREF_2)] | AAS | 4.3 ± 1.0 | 7.1 ± 1.2 |
| [[3](#_ENREF_3)] | ICP-MS | 1.33 ± 0.31 | 3.68 ± 0.51 |
| [[4](#_ENREF_4)] | PIXE | 1.5 | 2.64 ± 0.4 |
| [[5](#_ENREF_5)] | INAA | 0.24 ± 0.03 | 1.33 ± 0.20 |
| [[6](#_ENREF_6)] | INAA | 0.295 ± 0.047 | 0.472 ± 0.135 |
| [[7](#_ENREF_7)] | LA-ICP-MS | 0.16 ± 0.04 |  |
| [[8](#_ENREF_8)] | AAS | 0.26 ± 0.02 | 0.49 ± 0.03 |
| GP | | | |
| APS | XRF | 0.40 ± 0.05 | 1.53 ± 0.10 |
| [[3](#_ENREF_3)] | ICP-MS | 1.33 ± 0.31 | 5.93 ± 0.97 |
| [[4](#_ENREF_4)] | PIXE | 1.4 | 2.6 ± 0.4 |
| SN | | | |
| APS | XRF | 0.26 ± 0.22 | 1.21 ± 0.17 |
| [[7](#_ENREF_7)] | LA-ICP-MS | 0.30 ± 0.05 |  |
| [[9](#_ENREF_9)] | LA-ICP-MS | 0.423 ± 0.02 |  |

AAS: Atomic absorption spectroscopoy

ICP-MS: Inductively coupled plasma mass spectrometry

INAA: Instrumental neutron activation analysis

LA-ICP-MS: Laser Ablation Inductively Coupled Plasma Mass Spectrometry

PIXE: Proton induced x-ray emission

XRF: X-ray fluorescence

**References**

1. Zheng W, Jiang YM, Zhang YS, Jiang WD, Wang XQ, et al. (2009) Chelation therapy of manganese intoxication with para-aminosalicylic acid (PAS) in Sprague-Dawley rats. Neurotoxicology 30: 240-248.

2. Fitsanakis VA, Zhang N, Anderson JG, Erikson KM, Avison MJ, et al. (2008) Measuring brain manganese and iron accumulation in rats following 14 weeks of low-dose manganese treatment using atomic absorption spectroscopy and magnetic resonance imaging. Toxicol Sci 103: 116-124.

3. Morello M, Canini A, Mattioli P, Sorge RP, Alimonti A, et al. (2008) Sub-cellular localization of manganese in the basal ganglia of normal and manganese-treated rats - An electron spectroscopy imaging and electron energy-loss spectroscopy study. Neurotoxicology 29: 60-72.

4. Reaney SH, Bench G, Smith DR (2006) Brain accumulation and toxicity of Mn(II) and Mn(III) exposures. Toxicol Sci 93: 114-124.

5. Lai JCK, Chan AWK, Leung TKC, Minski MJ, Lim L (1992) Neurochemical changes in rats chronically treated with a high concentration of manganese chloride. Neurochem Res 17: 841-847.

6. Lai JCK, Minski MJ, Chan AWK, Lim L, Davison AN (1981) BRAIN REGIONAL MANGANESE DISTRIBUTION AFTER CHRONIC MANGANESE TREATMENT. Biochem Soc Trans 9: 228.

7. Matusch A, Depboylu C, Palm C, Wu B, Hoglinger GU, et al. (2010) Cerebral Bioimaging of Cu, Fe, Zn, and Mn in the MPTP Mouse Model of Parkinson's Disease Using Laser Ablation Inductively Coupled Plasma Mass Spectrometry (LA-ICP-MS). J Am Soc Mass Spectrom 21: 161-171.

8. Deskin R, Bursian SJ, Edens FW (1981) NEUROCHEMICAL ALTERATIONS INDUCED BY MANGANESE CHLORIDE IN NEONATAL RATS. Neurotoxicology 2: 65-73.

9. Hare DJ, George JL, Grimm R, Wilkins S, Adlard PA, et al. (2010) Three-dimensional elemental bio-imaging of Fe, Zn, Cu, Mn and P in a 6-hydroxydopamine lesioned mouse brain. Metallomics 2: 745-753.
