# Supplementary material for: X-Ray Fluorescence Imaging: A New Tool for Studying Manganese Neurotoxicity
Source: PLoS One. 2012 Nov 19;7(11):e48899. doi: 10.1371/journal.pone.0048899 (PMC3501493; doi:10.1371/journal.pone.0048899)
Supplement: Table S3 — Linear regression parameters for pixel scatter plots. (DOCX) [file pone.0048899.s010.docx]

**Table S3. Linear regression parameters for pixel scatter plots**

|  |  | Slope | CI | Intercept | Correlation |  | ANCOVA |  |
| --- | --- | --- | --- | --- | --- | --- | --- | --- |
|  |  | (μg_metal_/μg_Mn_) | (μg_metal_/μg_Mn_) | (μg_metal_/μg_Mn_) | r | p | F | p |
| Cortex | | | | | | | | |
| Cu vs Mn | C | 0.06 | (-0.12, 0.23) | 1.46 | 0.01 | 0.54 | 2.41 | 0.1204 |
|  | T | 0.18 | (0.13, 0.23) | 1.20 | 0.07 | < 0.01 |  |  |
| Fe vs Mn | C | 15.98 | (14.31, 17.65) | 5.93 | 0.30 | < 0.01 | 609.6 | < 0.001 |
|  | T | -6.07 | (-6.68, -5.45) | 14.29 | -0.20 | < 0.01 |  |  |
| Zn vs Mn | C | 3.14 | (2.78, 3.50) | 8.14 | 0.28 | < 0.01 | 16.05 | < 0.001 |
|  | T | 4.52 | (4.25, 4.79) | 5.75 | 0.33 | < 0.01 |  |  |
| Caudate putamen | | | | | | | | |
| Cu vs Mn | C | 0.25 | (0.10, 0.39) | 1.42 | 0.05 | < 0.01 | 16.16 | < 0.001 |
|  | T | -0.07 | (-0.11, -0.04) | 1.67 | -0.05 | < 0.01 |  |  |
| Fe vs Mn | C | 1.10 | (0.36, 1.84) | 11.01 | 0.04 | <0.01 | 2.49 | 0.1143 |
|  | T | 0.35 | (0.10, 0.59) | 11.45 | 0.03 | < 0.01 |  |  |
| Zn vs Mn | C | 1.24 | (0.85, 1.63) | 8.13 | 0.09 | < 0.01 | 12.51 | < 0.001 |
|  | T | 0.57 | (0.49, 0.65) | 8.41 | 0.17 | < 0.01 |  |  |
| Axon bundle | | | | | | | | |
| Cu vs Mn | C | 0.60 | (0.27, 0.94) | 0.88 | 0.09 | < 0.01 | 15.15 | < 0.001 |
|  | T | -0.16 | (-0.25, -0.08) | 1.22 | -0.08 | < 0.01 |  |  |
| Fe vs Mn | C | 4.37 | (1.60, 7.14) | 7.57 | 0.08 | < 0.01 | 1.92 | 0.1655 |
|  | T | 1.55 | (0.63, 2.47) | 7.92 | 0.07 | < 0.01 |  |  |
| Zn vs Mn | C | 2.78 | (1.83, 3.73) | 5.77 | 0.15 | < 0.01 | 19.06 | < 0.001 |
|  | T | 0.76 | (0.59, 0.93) | 5.80 | 0.18 | < 0.01 |  |  |
| Globus pallidus | | | | | | | | |
| Cu vs Mn | C | 0.31 | (0.03, 0.58) | 1.15 | 0.06 | 0.03 | 4.25 | 0.0393 |
|  | T | 0.049 | (0.01, 0.08) | 1.06 | 0.05 | < 0.01 |  |  |
| Fe vs Mn | C | 9.65 | (7.64, 11.67) | 10.42 | 0.27 | < 0.01 | 60.36 | < 0.001 |
|  | T | 1.10 | (0.78, 1.42) | 11.57 | 0.11 | < 0.01 |  |  |
| Zn vs Mn | C | 3.29 | (2.71, 3.86) | 6.59 | 0.32 | < 0.01 | 62.56 | < 0.001 |
|  | T | 0.65 | (0.55, 0.74) | 6.12 | 0.21 | < 0.01 |  |  |
| Substantial nigra | | | | | | | | |
| Cu vs Mn | C | 2.46 | (1.19, 3.00) | 0.61 | 0.30 | < 0.01 | 72.46 | < 0.001 |
|  | T | 0.05 | (-0.07, 0.18) | 1.32 | 0.02 | 0.41 |  |  |
| Fe vs Mn | C | 11.70 | (9.12, 14.29) | 8.00 | 0.30 | < 0.01 | 56.21 | < 0.001 |
|  | T | 1.25 | (0.63, 1.87) | 10.73 | 0.09 | < 0.01 |  |  |
| Zn vs Mn | C | 3.75 | (2.97, 4.52) | 5.34 | 0.32 | < 0.01 | 54.12 | < 0.001 |
|  | T | 1.38 | (1.26, 1.50) | 4.81 | 0.44 | < 0.01 |  |  |

C, control; CI, confidence interval; F, F-distribution value; p, probability of a randomly occurring result being greater than the observation; r, Pearson’s correlation coefficient; T, treated; T1, treated group 1; T2, treated group 2.
