# Supplementary material for: X-Ray Fluorescence Imaging: A New Tool for Studying Manganese Neurotoxicity
Source: PLoS One. 2012 Nov 19;7(11):e48899. doi: 10.1371/journal.pone.0048899 (PMC3501493; doi:10.1371/journal.pone.0048899)
Supplement: Table S4 — Linear regression parameters for brain region scatter plot. (DOCX) [file pone.0048899.s011.docx]

**Table S4. Linear regression parameters for brain region scatter plot**

|  |  | Slope | CI | Intercept | Correlation |  | ANCOVA |  |
| --- | --- | --- | --- | --- | --- | --- | --- | --- |
|  |  | (μg_metal_/μg_Mn_) | (μg_metal_/μg_Mn_) | (μg_metal_/μg_Mn_) | r | p | F | p |
| Cu vs Mn | C | 1.16 | (-0.78 , 3.09) | 0.97 | 0.41 | 0.21 | 1.63 | 0.22 |
|  | T1 | -0.04 | (-0.26, 0.18) | 1.21 | -0.12 | 0.70 |  |  |
|  | T2 | 0.07 | (-0.78, 0.92) | 1.48 | 0.12 | 0.83 |  |  |
| Fe vs Mn | C | 19.65 | (9.89, 29.41) | 5.84 | 0.84 | < 0.01 | 7.97 | <0.01 |
|  | T | 2.98 | (0.56, 5.41) | 8.04 | 0.52 | 0.02 |  |  |
| Zn vs Mn | C | 6.42 | (-4.05, 16.89) | 5.81 | 0.42 | 0.20 | 1.29 | 0.29 |
|  | T1 | 1.12 | (-2.24, 4.47) | 7.89 | 0.31 | 0.45 |  |  |
|  | T2 | 0.91 | (-0.22, 2.05) | 5.51 | 0.49 | 0.10 |  |  |

C, control; CI, confidence interval; F, F-distribution value; p, probability of a randomly occurring result being greater than the observation; r, Pearson’s correlation coefficient; T, treated; T1, treated group 1; T2, treated group 2.
